# A pipeline for automated deep learning liver segmentation (PADLLS) from contrast enhanced CT exams

**DOI:** 10.1038/s41598-022-20108-8

**Published:** 2022-09-22

**Authors:** Jayasuriya Senthilvelan, Neema Jamshidi

**Affiliations:** Department of Radiological Sciences, David Geffen School of Medicine, University of California, Los Angeles, 757 Westwood Ave, Suite 2125, Los Angeles, CA 90095 USA

**Keywords:** Diagnostic markers, Three-dimensional imaging, Tomography, Image processing, Machine learning

## Abstract

Multiple studies have created state-of-the-art liver segmentation models using Deep Convolutional Neural Networks (DCNNs) such as the V-net and H-DenseUnet. Oversegmentation however continues to be a problem. We set forth to address these limitations by developing a an automated workflow that leverages the strengths of different DCNN architectures, resulting in a pipeline that enables fully automated liver segmentation. A Pipeline for Automated Deep Learning Liver Segmentation (PADLLS) was developed and implemented that cascades multiple DCNNs that were trained on more than 200 CT scans. First, a V-net is used to create a rough liver, spleen, and stomach mask. After stomach and spleen pixels are removed using their respective masks and ascites is removed using a morphological algorithm, the scan is passed to a H-DenseUnet to yield the final segmentation. The segmentation accuracy of the pipleline was compared to the H-DenseUnet and the V-net using the SLIVER07 and 3DIRCADb datasets as benchmarks. The PADLLS Dice score for the SLIVER07 dataset was calculated to be 0.957 ± 0.033 and was significantly better than the H-DenseUnet’s score of 0.927 ± 0.044 (*p* = 0.0219) and the V-net’s score of 0.872 ± 0.121 (*p* = 0.0067). The PADLLS Dice score for the 3DIRCADb dataset was 0.965 ± 0.016 and was significantly better than the H-DenseUnet’s score of 0.930 ± 0.041 (*p* = 0.0014) the V-net’s score of 0.874 ± 0.060 (*p* < 0.001). In conclusion, our pipeline (PADLLS) outperforms existing liver segmentation models, serves as a valuable tool for image-based analysis, and is freely available for download and use.

## Introduction

The growth and development of deep learning applications in biomedical imaging research have been profound in the past decade. To leverage these developments and to fully achieve goals of radiomic and radiogenomic objectives, fully automated organ segmentation will become increasingly important. The liver is a critical organ in health and disease, notably in oncologic disease (primary and metastatic) as well as endocrine and metabolic disorders, with computed tomography (CT) imaging playing a critical role for diagnosis, treatment planning, and follow-up for numerous hepatic diseases^1–3^. Segmentation applications have increased dramatically, but growth has been limited by the widespread availability of tools and semi-automated approaches that require user input. Although these methods give physicians greater control over the nature of the segmentation, they are also subjective and time-consuming. As a result, there is a need for automated segmentation for advancing quantitative analyses of livers.

Traditional approaches for organ segmentation can be classified into three groups: region-based methods, classification and clustering methods, and hybrid methods^4^. Region-based methods include thresholding and region growing^5^. Disadvantages of these two methods include sensitivity to noise and segmentation accuracy dependence on the operator's seed point selection, respectively. Classification methods, like k-Nearest Neighbor (kNN) and Maximum Likelihood Estimation, typically classify each pixel one at a time based on the training data. Clustering methods are similar to classification models with the exception that they do not require training data, including, for example, K-means and Expectation Maximization. The drawback to this category is the inability to factor in extensive spatial information^6^. Hybrid methods are based on both the region of interest (ROI) and boundary information (calculating a gradient based on pixel values) but are hindered by the requisite user interaction for every slice that is segmented in a series^7^.

Deep Convolutional Neural Networks (DCNNs) address many of the issues posed by earlier segmentation models. For instance, they can take into account both 2D and 3D spatial information. DCNNs are also highly adaptable, which is critical for liver segmentation as liver size, shape, and density vary widely from patient to patient. Additionally, once trained the method is fully automatic, so no user input is required^8^. Conversely, there are drawbacks to DCNNs, such as the large amount of manually segmented training data, time, and computational power it takes to train the model. Running the model successfully also requires significant GPU resources and time. However, the success of DCNNs in image segmentation challenges like LiTS (Liver Tumor Segmentation) justifies the initial investment of time and resources^9^.

Current state of the art liver segmentation DCNNs, such as the V-net from Gibson et al. and the H-DenseUnet from Li et al.^10, 11^, have overcome many of the challenges related to liver segmentation such as separation from potential structures such as the heart and kidney as well as minimizing the confounding effect of diaphragmatic motion. However, there remain limitations to the results from each of these models, notably oversegmentation of the stomach, spleen, and/or ascites. In this paper, we define oversegmentation as the inclusion of non-hepatic voxels in the final liver mask due to indistinct anatomic boundaries between organs and structures. These types of problems increase the false positive error and compromise the validity of any subsequent analyses.

In order to solve this issue, we developed a robust, automated pipeline approach by cascading the V-net and H-DenseUnet and applying knowledge-based heuristics; the result is a Pipeline for Automated Deep Learning Liver Segmentation (PADLLS). The V-net’s spleen and stomach masks were used to modify the original CT volume to entirely remove all spleen and stomach voxels. Ascites was also removed from the dataset through thresholding and morphological processing. The resulting edited volume was passed to the H-DenseUnet to generate the final liver segmentation. Testing on benchmark datasets (3DIRCADb and SLIVER07) demonstrated that our approach outperforms the H-DenseUnet and V-net, which is indicative of a synergistic improvement in accuracy. A direct comparison of our pipeline to other state-of-the-art models supports these findings.

## Methods

### Preprocessing

Data from both SLIVER07 (Segmentation of the Liver Competition 2007)^12^ and 3DIRCADb^13^ are available in a different orientation and file format than that which the V-net, H-DenseUnet, and our segmentation pipeline required. Hence, each slice of SLIVER07 and 3DIRCADb CT scans was rotated clockwise by 90° and reflected about the vertical axis. All datasets were then converted from either DICOM or MHD to NIfTI, which is the format used by all networks in this manuscript. This was accomplished by extracting the raw data array from the DICOM or MHD file and creating an entirely new NIfTI file.

### Test datasets

**Figure 1:**
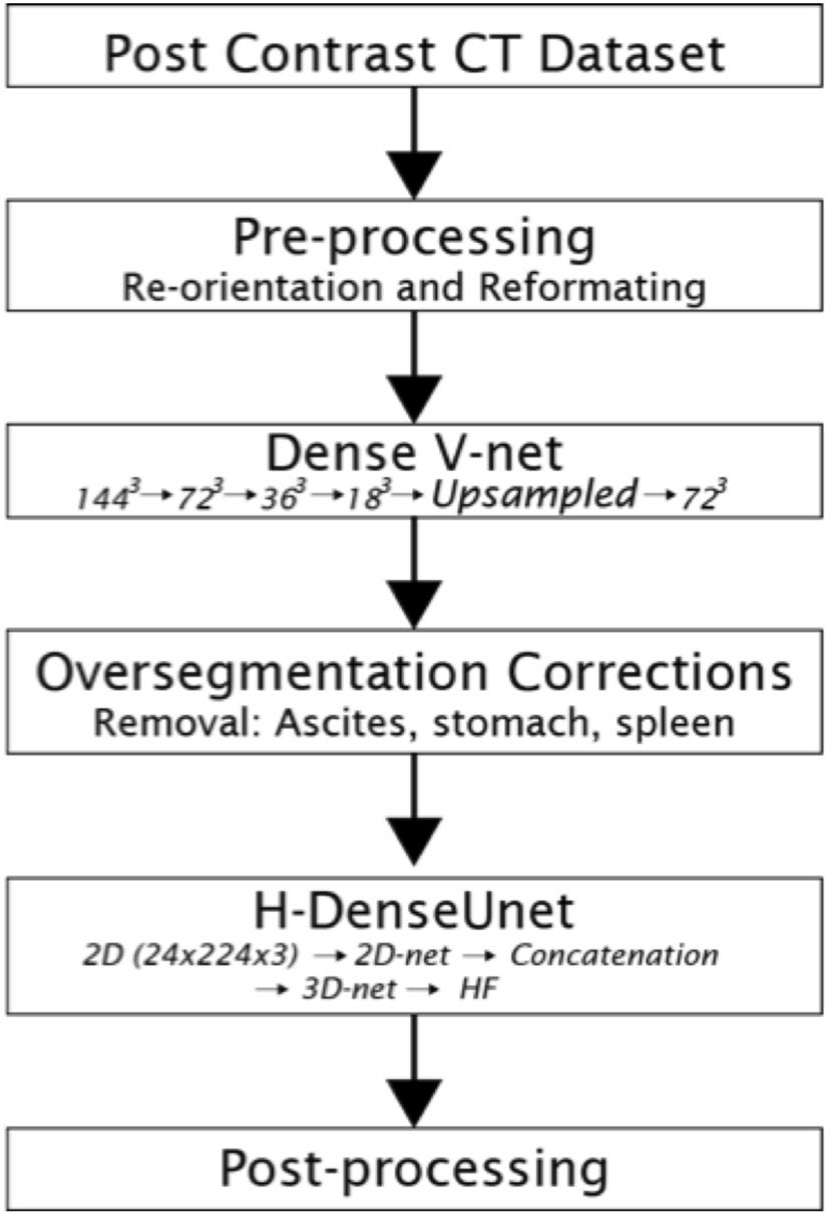
Flowchart schematic outlining the PADLLS steps. First, the imported images are standardized with respect to orientation and format. Next, the V-net segments the study for the initial liver mask (in addition to the stomach and spleen). Oversegmentation correction is then applied though thresholding and finally the H-DenseUnet further refines the liver segmentation.

The final liver segmentation pipeline in Fig. 1 was tested on two public challenge CT datasets: SLIVER07 and 3DIRCADb^13^. Most of the scans in the SLIVER07 datasets were of diseased livers, with cysts and tumors. All images were also contrast enhanced. The pipeline was run on an NVIDIA Quadro RTX4000 with 8 GB memory and a NVIDIA Tesla V100 with 32 GB memory. The SLIVER07 challenge provided 20 datasets in RAW and MHD format. These datasets were first converted to DICOM format before running them through the segmentation pipeline. Reference segmentations for each respective dataset were provided. The 3DIRCADb dataset with 20 contrast enhanced CT-scans (10 men and 10 women) and reference ‘gold standard’ manual segmentation was used as well. Seventy-five percent of the datasets in 3DIRCADb had hepatic tumors. Three additional datasets (multiphase CT scans) were used to test the effectiveness of ascites removal.

### Initial V-net segmentation

The initial V-net segmentation was performed using the Dense V-net proposed by Gibson et al.^10^. A summary of the network architecture is provided as follows. A 144 × 144 × 144 initial volume is provided as input to the network and convolutional downsampling is performed. The downsampled volume is then run through a series of dense feature stacks and convolutions, which are used to create activation maps at three resolutions. These maps are bilinearly upsampled, concatenated, and convolved to generate likelihood logits. Finally, an explicit spatial prior was added to these logits to generate the segmentation. The network was trained to segment eight different organs: pancreas, esophagus, duodenum, stomach, liver, spleen, left kidney, and gallbladder. The data used to train this network originated from the Cancer Imaging Archive Pancreas-CT dataset^14^ and Beyond the Cranial Vault (BTCV) Segmentation Challenge^15^. The V-net was trained using the following probabilistic Dice score function to calculate an L2 regularization loss.

1 $${\varvec{pDice}}_{{\varvec{l}}} \left( {{\varvec{L}}_{{\varvec{l}}}^{\prime \prime } ,{\varvec{R}}_{{\varvec{l}}} } \right) = \overline{{\left( {\frac{{{\text{min}}\left( {{\varvec{L}}_{{\varvec{l}}}^{\prime \prime } ,0.9} \right)}}{{\left\| {{\varvec{R}}_{{\varvec{l}}} } \right\|_{2} + \left\| {{\text{min}}\left( {{\varvec{L}}_{{\varvec{l}}}^{\prime \prime } ,0.9} \right)} \right\|_{2} }}} \right)}}$$

$L$ is the logit result of the V-net with nine different classes. $L$ becomes $L^{\prime }$ upon the addition of a spatial prior called $P$, introduced by Gibson in a previous work^16^. $L_{l}^{\prime \prime }$ is the result of applying the softmax function to $L^{\prime }$ for some label $l$. $R_{l}$ is the ground truth segmentation for some organ $l$. This network was trained for 6 h on a Titan X Pascal GPU with 12 GB of RAM. The liver segmentation from the V-net was used to define a bounding box for the subsequent H-DenseUnet. Further, using the stomach and spleen masks from the V-net, the stomach and spleen voxels were replaced with − 100 Hounsfield Units (HU) in the CT data passed to the H-DenseUnet in order to prevent any potential oversegmentation by the H-DenseUnet phase of the pipeline. Full architecture details of V-net can be found in Supplementary Table S1.

### Ascites correction

Since the H-DenseUnet and V-net were both observed to overestimate liver volumes in imaging studies with ascites, we felt it was important to correct for this. Hence, a 3D binary mask of all pixels less than 15 HU was created (inclusive of ascites and peritoneal fat; see Statistics and Analysis). Then, image opening was performed with a disk of radius 2 pixels on each slice. Image opening is the erosion of an image followed by dilation using a structuring element. Spherical and circular structural elements were used throughout this paper because they best preserve the border contours of the binary image. It is intended to remove small binary objects. It can be described with the following formula:

2 $${\varvec{A}} \circ {\varvec{B}} = \left( {{\varvec{A}}{ \ominus }{\varvec{B}}} \right) \oplus {\varvec{B}}$$

Here, A is the binary image and B is the structuring element. $\oplus$ and $\ominus$ refer to image dilation and image erosion, respectively. Overall, these steps eliminated any parts of the liver or other organs that were lower than 15 HU in intensity. Subsequently, a binary area filter selecting objects that were greater in area than 1500 pixels was applied to each image in the volumetric dataset. Finally, all CT dataset voxels that were included in this binary volume were set to − 100 HU in order to exclude ascites.

### Final H-DenseUnet segmentation

The output of the V-net segmentation was passed to the H-DenseUnet presented by Li et al.^11^. This network consisted of 3 critical components: a 2D DenseUnet, a 3D DenseUnet, and a Hybrid Fusion (HF) layer. The 2D DenseUnet is good at recognizing intra-slice features but fails to take into account information along the z-axis, whereas the 3D DenseUnet is good at recognizing inter-slice features but has a large computational cost. As a result, combining these two networks in a cascaded learning approach was determined to produce optimal segmentation results.

The H-DenseUnet was trained using a weighted cross-entropy loss function, seen below:

3 $${\varvec{L}}\left( {{\varvec{y}},\hat{\user2{y}}} \right) = - \frac{1}{{\varvec{N}}}\mathop \sum \limits_{{{\varvec{i}} = 1}}^{{\varvec{N}}} \mathop \sum \limits_{{{\varvec{c}} = 1}}^{3} {\varvec{w}}_{{\varvec{i}}}^{{\varvec{c}}} {\varvec{y}}_{{\varvec{i}}}^{{\varvec{c}}} {\text{log}}\widehat{{{\varvec{y}}_{{\varvec{i}}} }}^{{\varvec{c}}}$$

${w}_{i}^{c}$ represents the weight and ${y}_{i}^{c}$ is the ground truth for pixel $i$. ${\widehat{{y}_{i}}}^{c}$ is the probability that a pixel $i$ is found in class $c$, where the classes are background, lesion, and liver. This network was trained for 30 h using two NVIDIA Titan Xp GPUs (12 GB each).

First, the initial liver segmentation from the V-net was used to define a rough bounding box in the CT data, which was then resized to 224 × 224 × 12. Next, every three adjacent slices in the input volume (224 × 224 × 3) were passed to the 2D DenseUnet. These 2D segmentation results were concatenated with the 3D input volume (224 × 224 × 12) and fed into the 3D DenseUnet. Then, the HF layer was used to fuse the intra-slice and inter-slice features from the 2D DenseUnet and the 3D DenseUnet, respectively, to create a final liver segmentation. Full details of H-DenseUnet architecture can be found in Supplementary Table S2.

### Post-processing

The result of the H-DenseUnet segmentation was truncated to only include slices that had liver pixels in them. Sometimes the liver segmentation included lung pixels near the hepatic dome. Hence, all pixels in the liver binary mask with values less than 0 HU were excluded from the final segmentation. Volume filtering was performed to select the largest object and image closing was performed with disk of radius 2 pixels. Liver and tumor masks from the H-DenseUnet were combined into one liver binary volume. Any holes in the mask were filled, and image closing was performed on the volume with a sphere of radius 3 pixels. Image closing is defined as the dilation of a binary image followed by erosion using a structuring element, intended to fill small holes in a binary image. It can be described with the following formula:

4 $${\varvec{A}} \cdot {\varvec{B}} = \left( {{\varvec{A}} \oplus {\varvec{B}}} \right){ \ominus }{\varvec{B}}$$

A is the binary image and B is the structuring element. $\oplus$ and $\ominus$ retain their meaning from Eq. (2). Schematic of full segmentation pipeline from start to finish can be seen in Fig. 1.

### Statistics and analysis

For the ascites correction, a receiver operating characteristic (ROC) curve was generated across the range of 0 to 30 HU, with increments of 1 HU between 10 and 20 HU and increments of 5 HU otherwise. Volumetric Overlap Error (VOE), Relative Volume Difference (RVD), Average Symmetric Surface Distance (ASD), Root Mean Square Symmetric Surface Distance (RMSD), Hausdorff Distance (HD), and Dice score were calculated as metrics to comprehensively compare the segmentation results of the different algorithms. In the following formulas, $A$ is the binary segmentation result and $B$ is the ground truth mask. $d$ is the Euclidean distance between two points. $S\left(A\right)$ and $S\left(B\right)$ are the surface of the ground truth mask and binary segmentation result, respectively.

Dice coefficients for the segmentation results were calculated as:

5 $${\varvec{Dice}} = 2\left( {{\varvec{A}} \cap {\varvec{B}}} \right)/\left( {\left| {\varvec{A}} \right| + \left| {\varvec{B}} \right|} \right)$$

A Dice score of 1 reflects perfect segmentation of the entire study. VOE was calculated as a percent, where a VOE of 0% means a perfect segmentation. VOE was also used to compare the effect of ascites on the performance of the V-net and H-DenseUnet with our pipeline. The VOE was calculated as:

6 $${\varvec{VOE}} = 100\left( {1 - \left( {\left| {{\varvec{A}} \cap {\varvec{B}}} \right|} \right)/\left| {{\varvec{A}} \cup {\varvec{B}}} \right|} \right)$$

RVD was also calculated with the following formula:

7 $${\varvec{RVD}} = 100\left( {\left( {\left| {\varvec{A}} \right| - \left| {\varvec{B}} \right|} \right)/\left| {\varvec{B}} \right|} \right)$$

An RVD of 0% means a perfect segmentation. ASD was calculated in millimeters with the following formula:

8 $${\varvec{ASD}}\left( {{\varvec{A}},{\varvec{B}}} \right) = \frac{1}{{\left| {{\varvec{S}}\left( {\varvec{A}} \right)} \right| + \left| {{\varvec{S}}\left( {\varvec{B}} \right)} \right|}} \times \left( {\mathop \sum \limits_{{{\varvec{s}}_{{\varvec{A}}} \in {\varvec{S}}\left( {\varvec{A}} \right)}} {\varvec{d}}\left( {{\varvec{s}}_{{\varvec{A}}} ,{\varvec{S}}\left( {\varvec{B}} \right)} \right) + \mathop \sum \limits_{{{\varvec{s}}_{{\varvec{A}}} \in {\varvec{S}}\left( {\varvec{A}} \right)}} {\varvec{d}}({\varvec{s}}_{{\varvec{B}}} ,{\varvec{S}}\left( {\varvec{A}} \right)} \right)$$

For all points on the surface of volume A, the Euclidean distance is calculated to the nearest surface point on volume B. This process is repeated for all surface points on volume B with respect to the nearest surface point on volume A. The mean of these distances yields the ASD. An ASD of 0 mm means a perfect segmentation. The RMSD in millimeters is calculated as follows:

9 $${\varvec{RMSD}}\left( {{\varvec{A}},{\varvec{B}}} \right) = \frac{1}{{\left| {{\varvec{S}}\left( {\varvec{A}} \right)} \right| + \left| {{\varvec{S}}\left( {\varvec{B}} \right)} \right|}} \times \sqrt {\mathop \sum \limits_{{{\varvec{s}}_{{\varvec{A}}} \in {\varvec{S}}\left( {\varvec{A}} \right)}} {\varvec{d}}^{2} \left( {{\varvec{s}}_{{\varvec{A}}} ,{\varvec{S}}\left( {\varvec{B}} \right)} \right) + \mathop \sum \limits_{{{\varvec{s}}_{{\varvec{A}}} \in {\varvec{S}}\left( {\varvec{A}} \right)}} {\varvec{d}}^{2} ({\varvec{s}}_{{\varvec{B}}} ,{\varvec{S}}\left( {\varvec{A}} \right)}$$

It is simply the root mean square of the distances calculated during the process of calculating ASD. An RMSD of 0 mm means a perfect segmentation. Hausdorff distance was calculated in millimeters with the following formula:

10 $${\varvec{HD}}\left( {{\varvec{A}},{\varvec{B}}} \right) = \mathop {{\text{max}}}\limits_{{{\varvec{a}} \in {\varvec{A}}}} \left\{ {\mathop {{\text{min}}}\limits_{{{\varvec{b}} \in {\varvec{B}}}} \left\{ {{\varvec{d}}\left( {{\varvec{a}},{\varvec{b}}} \right)} \right\}} \right\}$$

It is defined as the largest distance between the surface of A to the closest point in the surface of B. A Hausdorff distance of 0 mm means a perfect segmentation.

Statistical significance criterion was defined as *p* values $<$ 0.05 with Welch’s t-test.

UCLA Institutional Review Board approval was obtained (IRB#: 10-001869) and included waiver of informed consent. The authors attest they are in compliance with human studies committees of the authors’ institutions and performed in accordance with the ethical standards as laid down in the 1964 Declaration of Helsinki and its later amendments or comparable ethical standards.

### Data availability

The SLIVER07 dataset analyzed in this study is available at https://sliver07.grand-challenge.org. The 3DIRCADb dataset analyzed in this study is available at https://www.ircad.fr/research/data-sets/liver-segmentation-3d-ircadb-01/. The entire code base and dockerfile for the code environment in Python are provided at https://github.com/neemajamshidi/PADLLS and is also available as a Docker image on Docker Hub at jaysen20/siml-liver-net.

## Results

### Component-wise validation

The validation of the individual components of the liver segmentation pipeline were recorded in their respective papers; the V-net used in the initial segmentation was trained with more than 90 datasets^10^ and the H-DenseUnet that was used was trained with more than 130 datasets^11^. Hence, the pipeline in this paper has been trained on more than 220 abdominal CT scans. The average Dice score per case for the H-DenseUnet was 0.961 for the LiTS challenge^9, 11^. On a multi-center dataset with 90 subjects, the average Dice score per case for the V-net was 0.95 for the liver^10^. While these results are encouraging, each of the networks has limitations that can result in oversegmentation of the liver, for example by inappropriate inclusion of abdominal ascites or other (non-hepatic) abdominal organs as part of the liver mask.

### Ascites correction

**Figure 2:**
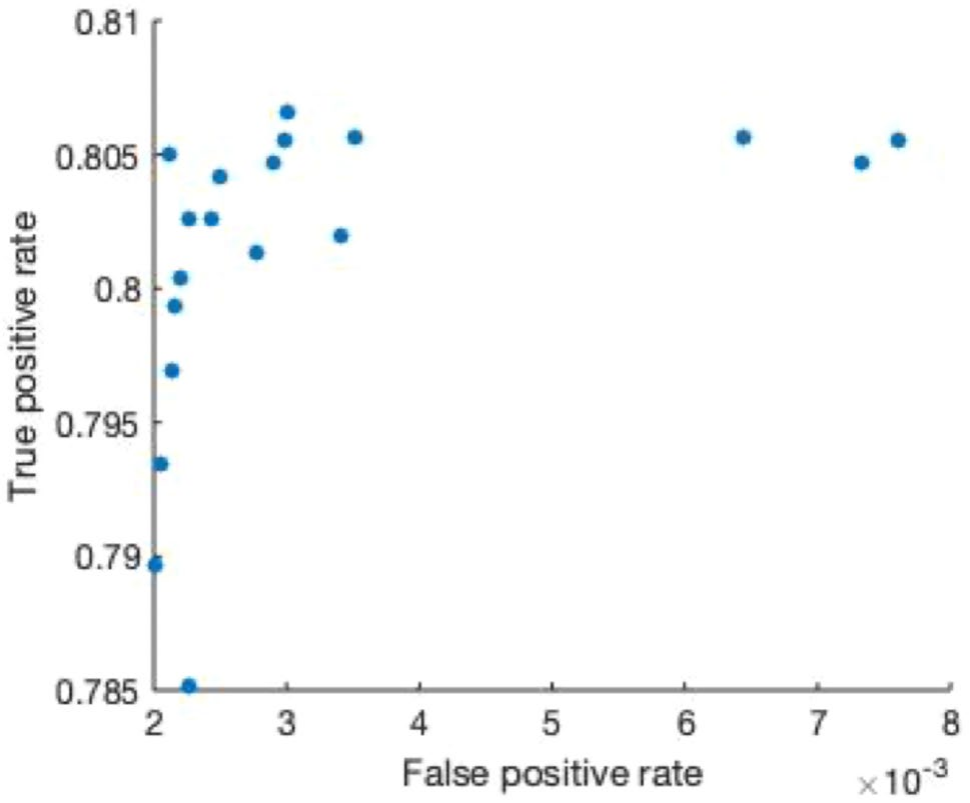
ROC curve for the ascites correction threshold. The threshold value was varied from 0 to 50 HU in increments of 5 HU to construct this curve. From 10 to 20 HU, the threshold was varied in increments of 1 HU. The optimal value of 15 HU was selected as the ascites threshold because it maximizes true positive rate and minimizes false positive rate.

**Figure 3:**
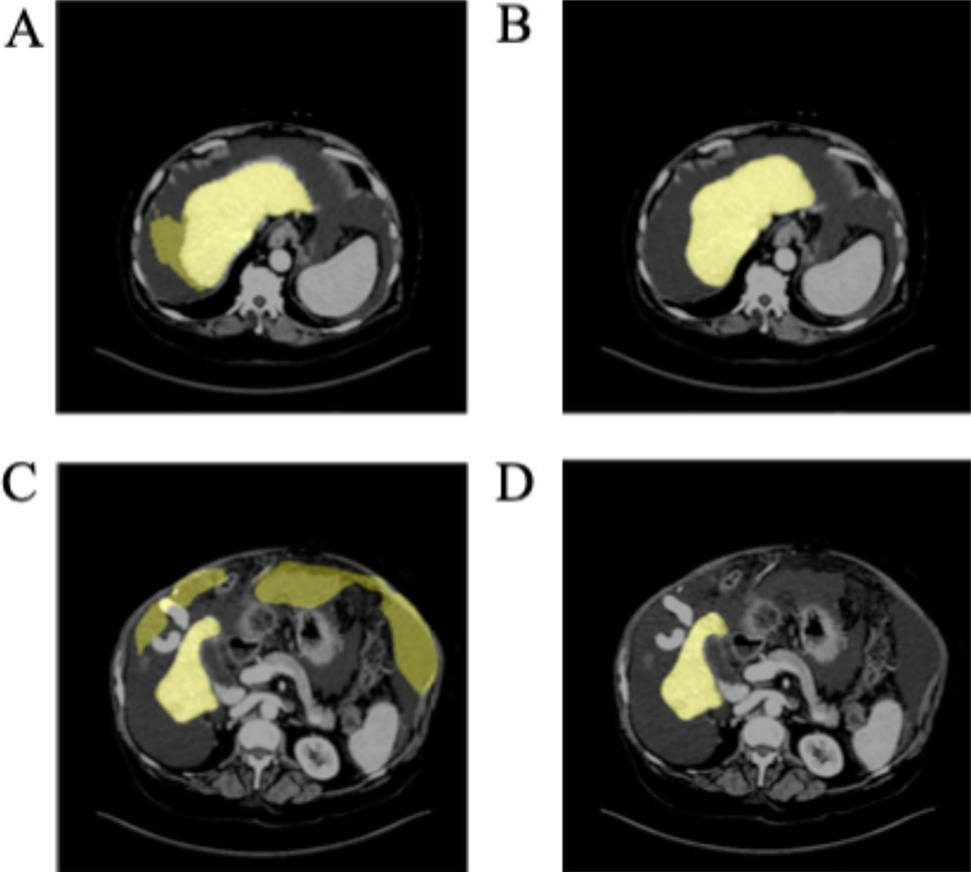
Example of PADLLS oversegmentation correction of ascites. (**A**) V-net liver segmentation with no ascites correction and (**C**) H-DenseUnet segmentation with no ascites correction. (**B**) and (**D**) are final pipeline segmentations with ascites correction. The yellow shading in all of the images demarcates the liver. Window width (WW) and window length (WL) of 30 and 150 HU were used, respectively.

ROC analysis was used to identify an optimal threshold to remove ascites (Fig. 2). The ascites cutoff was selected at peak of the curve, 15 HU, a value that is consistent with the typical CT attenuation range of 5 to 15 HU for simple fluid. An example of the effects of the ascites correction for each of the individual DCNNs versus the pipeline are illustrated in Fig. 3. Figure 3A and C show that the V-net and H-DenseUnet have erroneously included abdominal fluid in their liver segmentations. However, once the ascites correction is applied, the liver segmentation contour becomes more accurate, as shown in Fig. 3B and D. The relative oversegmentation of ascites by these networks was quantified by comparing their respective VOE% for the case in Fig. 3. These results are reported in Table 3, which reveals that PADLLS decreases ascites oversegmentation by approximately a factor of two and five for the V-net and H-DenseUnet, respectively.

A quantitative assessment of the ascites correction was performed through comparison of the Dice score with and without the correction. For 3DIRCADb, the average Dice improved from 0.926 to 0.965, a statistically significant difference based on Welch’s t-test (*p* = 0.0008). For SLIVER07, the average Dice improved from 0.940 to 0.957, but this difference was not statistically significant (*p* = 0.170). Given the relatively small number of datasets in 3DIRCADb and SLIVER07, this difference may reflect a higher incidence of ascites in 3DIRCADb than SLIVER07. Regardless, we expect that correction of ascites may have a significant impact on potential applications for liver segmentation (e.g. tumor segmentation, radiomic studies, etc.).

### Correction abdominal organ oversegmentation

**Figure 4:**
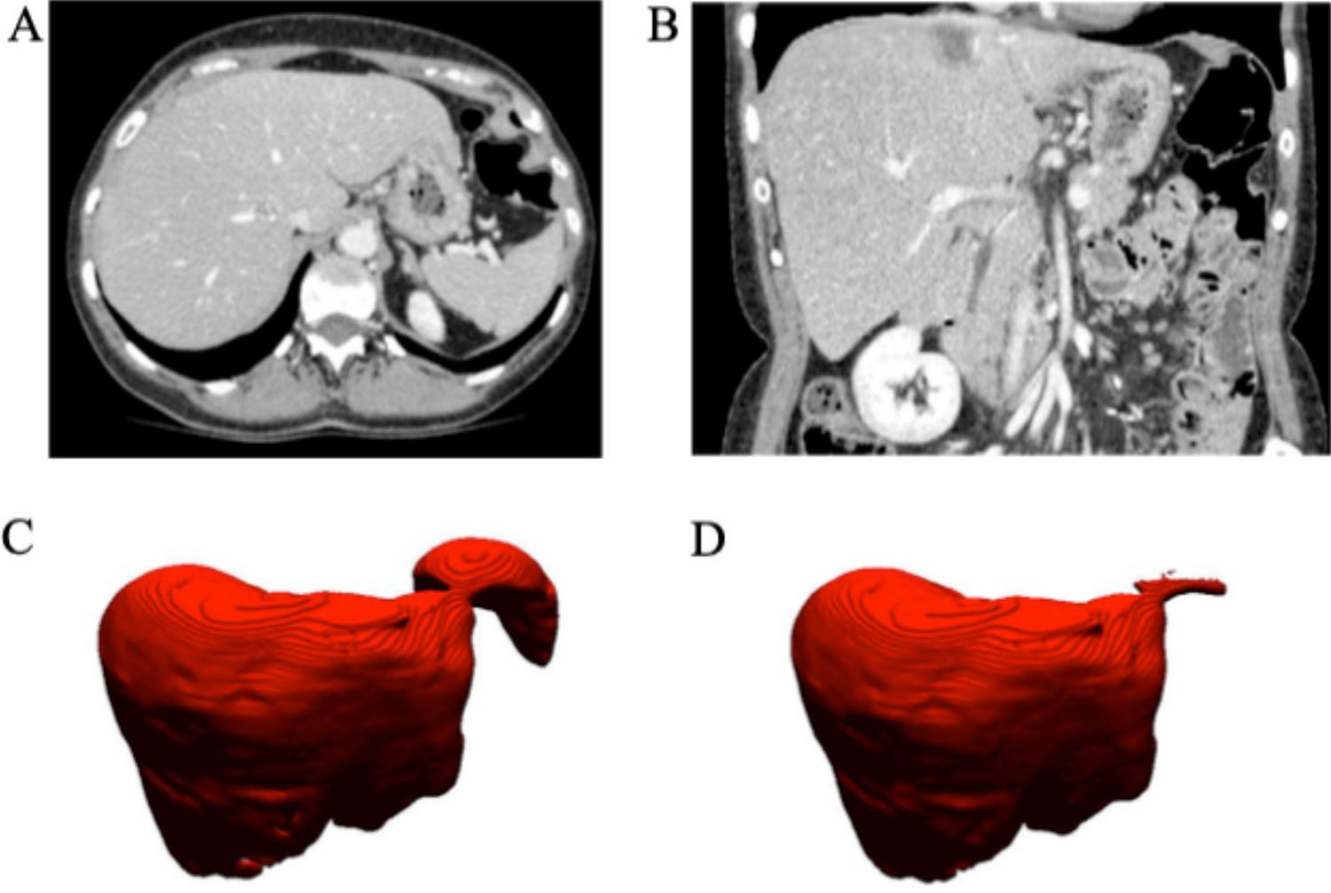
Example of PADLLS oversegmentation correction of the spleen and stomach. Axial (**A**) and coronal (**B**) CT slices of the segmented dataset at the level of the liver. (**C**) H-DenseUnet segmentation of liver from 3DIRCADb dataset that includes the spleen. (**D**) Final pipeline segmentation that accurately captures the lateral margin of the liver adjacent to the stomach but does not oversegment with the stomach or spleen. WW and WL of 350 and 40 HU were used, respectively.

The H-DenseUnet frequently oversegments by including portions of liver-adjacent organs (Fig. 4). Figure 4A and B are coronal and axial slices of the liver from a single example dataset used to demonstrate this phenomenon. Figure 4C shows an example 3D liver segmentation volume performed by the H-DenseUnet that has erroneously included a large portion of the spleen. Our pipeline, however, solves this issue by removing the spleen (identified from the V-net segmentation) before feeding this input into the H-DenseUnet. Figure 4D shows a 3D model of the liver from the same CT scan following PADLLS segmentation, notably without oversegmentation of the spleen or other abdominal organs.

### Pipeline validation

**Figure 5:**
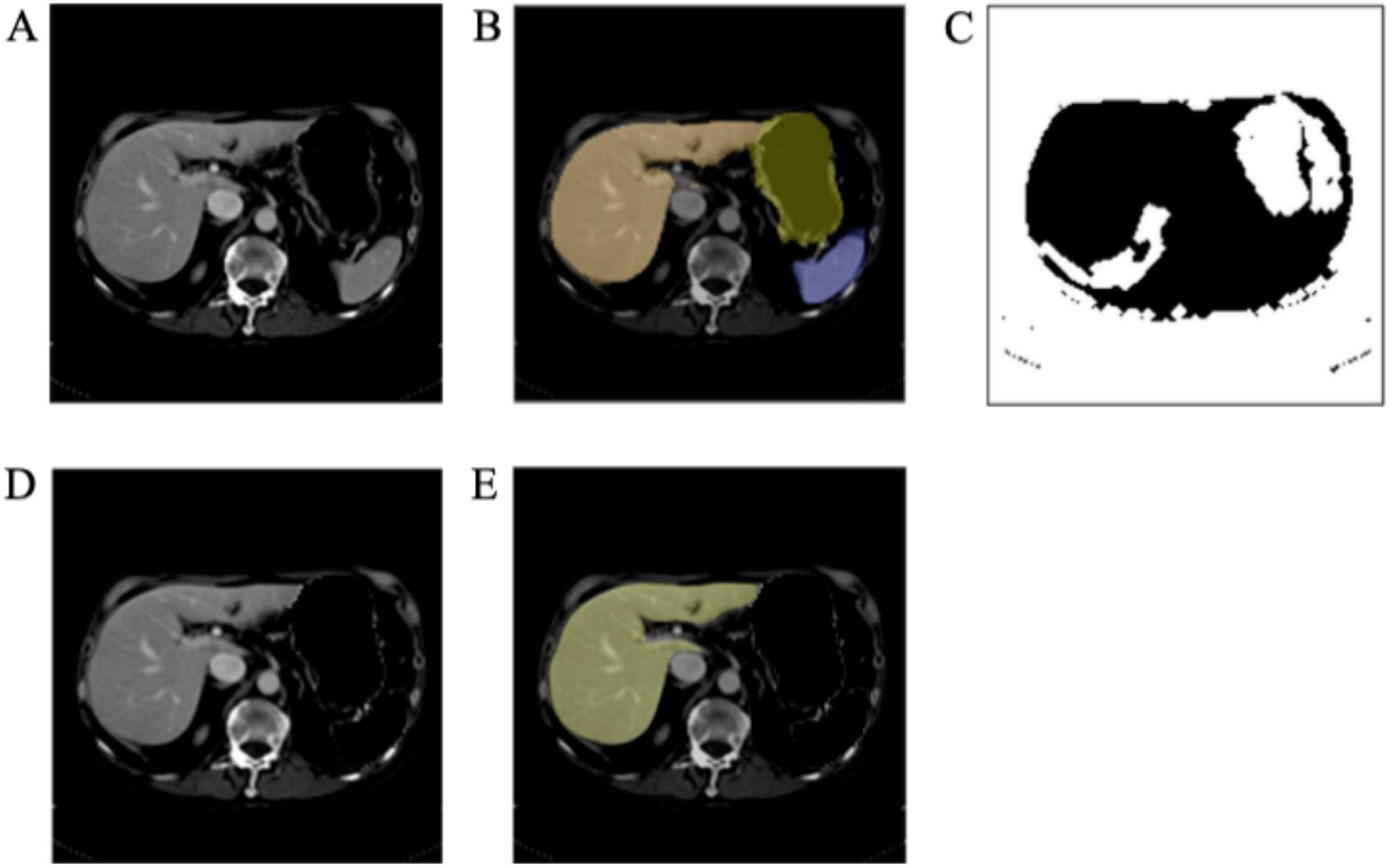
Example outlining intermediate pipeline steps. (**A**) An axial slice of a post-contrast CT image through the middle of the liver. (**B**) Initial result following V-net segmentation. Liver, stomach, and spleen are shaded orange, yellow, and purple, respectively. (**C**) Filter mask for ascites removal. (**D**) Input to the H-DenseUnet. Note that the stomach, spleen, and ascites pixels have been replaced with − 100 HU. (**E**) Final pipeline segmentation result with liver shaded in red. WW and WL are 400 and 50 for all of CT scans.

Another exemplar illustrating the step wise improvement in segmentation during the intermediate steps is shown in Fig. 5. Figure 5A shows a preprocessed, unsegmented, axial CT slice. Figure 5B shows the V-net segmentation of the liver, stomach, and spleen. Figure 5C shows the result of the heuristic method of creating a binary ascites mask. Figure 5D was created by setting all stomach, spleen, and ascites pixels to − 100 HU using the yellow and purple segmentations from Fig. 5B and the ascites mask from Fig. 5C. Figure 5D then became the input for the H-DenseUnet. The final pipeline segmentation of the liver is provided in Fig. 5E.

**Figure 6:**
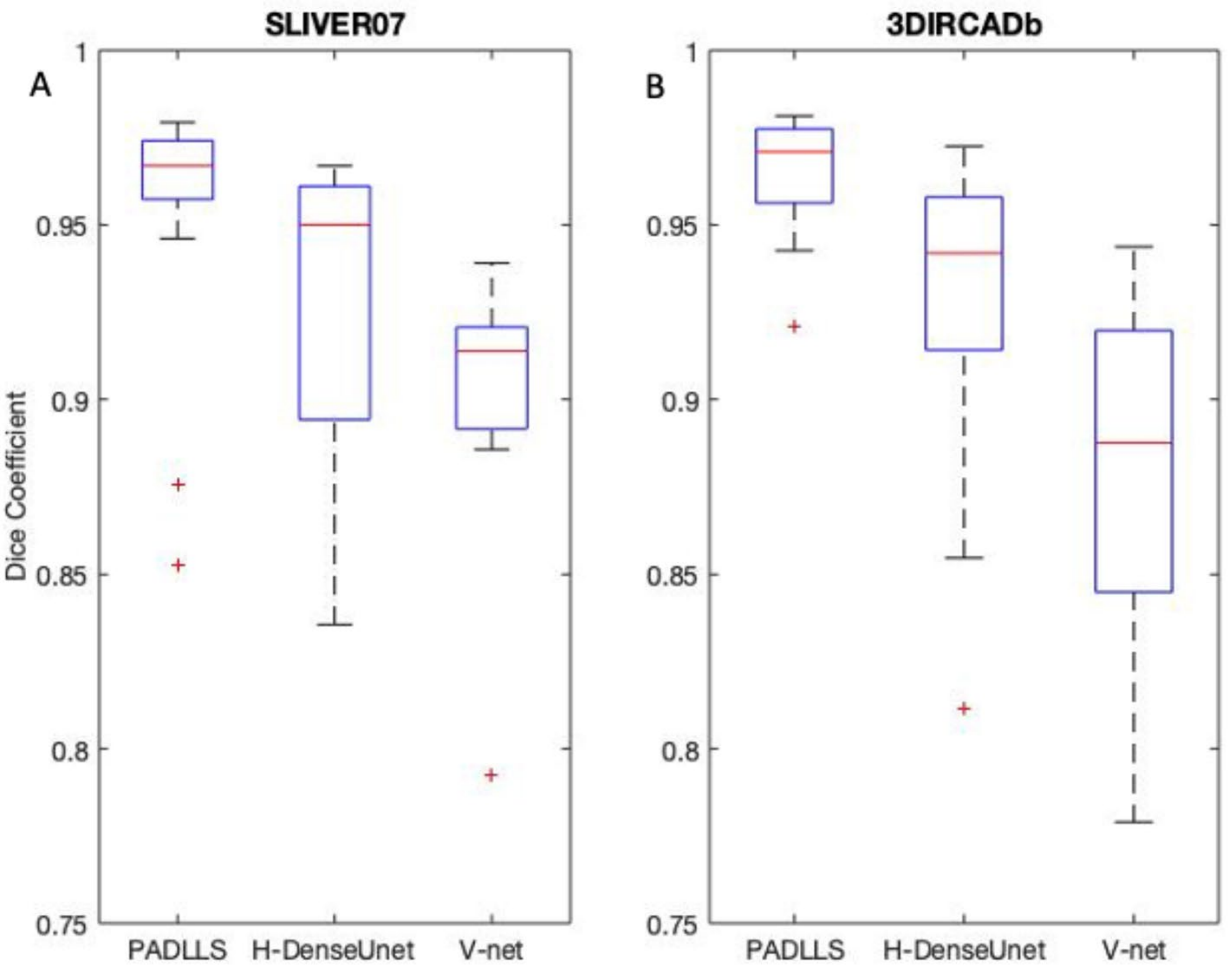
Statistical summary of the Dice coefficients for SLIVER07 (**A**) and 3DIRCADb (**B**) for our pipeline, H-DenseUnet, and V-net corresponding to left, middle, and right boxplots, respectively. Box and whisker plots with blue boxes showing the interquartile range, red line indicating the median, and red pluses indicating outliers. 3DIRCADb and SLIVER07 consist of 2823 and 4159 individual CT slices, respectively.

The validation of the proposed liver segmentation pipeline was performed using the 3DIRCADb and SLIVER07 challenge datasets. The average Dice score for our pipeline for 3DIRCADb was 0.965 $\pm$ 0.016. The average Dice score for our pipeline for the SLIVER07 challenge was 0.957 $\pm$ 0.033. Tables 1 and 2 compare the average PADLLS Dice score to those of the V-net and H-DenseUnet for the SLIVER07 and 3DIRCADb benchmarks, respectively. From these tables we can see that PADLLS outperforms its constituent networks by a statistically significant margin (*p* $<$ 0.05) for both benchmarks. Thus we believe our pipeline leverages strengths of different types of DCNN and further improves upon them in a synergistic fashion, as reflected by the boxplots of Dice scores for each network in each validation dataset (Fig. 6). Not only is the segmentation accuracy of PADLLS higher than both H-DenseUnet and V-net (Tables 1 and 2), but the segmentation error is also lower, suggesting greater consistency with PADLLS segmentation (Table 3).

**Table 1:** Comparison of liver segmentation results on SLIVER07.

Model	Year	VOE (%)	RVD (%)	ASD (mm)	RMSD (mm)	HD (mm)	Dice	*p* value
V-net^10^	2018	21.15 $\pm$ 14.65	− 0.17 $\pm$ 0.16	3.55 $\pm$ 2.61	3.70 $\pm$ 2.27	83.90 $\pm$ 156.93	0.872 $\pm$ 0.121	0.0067*
H-DenseUnet^11^	2018	13.29 $\pm$ 7.38	0.0754 $\pm$ 0.1174	5.34 $\pm$ 8.13	6.85 $\pm$ 5.84	93.35 $\pm$ 131.38	0.927 $\pm$ 0.044	0.0219*
PADLLS	2022	8.14 $\pm$ 5.67	− 0.0056 $\pm$ 0.0812	1.72 $\pm$ 2.90	2.89 $\pm$ 5.26	33.63 $\pm$ 38.97	0.957 $\pm$ 0.033	–

An asterisk indicates a statistically significant difference in Dice score between our pipeline and another model (*p* < 0.05).

**Table 2:** Comparison of liver segmentation results on 3DIRCADb.

Model	Year	VOE (%)	RVD (%)	ASD (mm)	RMSD (mm)	HD (mm)	Dice	*p* value
V-net^10^	2018	21.85 ± 8.90	− 0.1726 ± 0.100	4.07 ± 2.05	4.07 ± 2.81	49.59 ± 49.40	0.874 ± 0.060	< 0.001*
H-DenseUnet^11^	2018	12.87 ± 6.87	0.0313 ± 0.1187	4.10 ± 4.63	5.89 ± 7.16	53.60 ± 43.69	0.930 ± 0.041	0.0014
PADLLS	2022	6.66 ± 2.89	− 0.0421 ± 0.033	1.31 ± 0.83	1.94 ± 1.85	29.73 ± 19.90	0.965 ± 0.016	–

An asterisk indicates a statistically significant difference in Dice score between our pipeline and another model (*p* < 0.05).

**Table 3:** Comparison of Volumetric Overlap Errors (VOE) for the case in Fig. 3.

Model	VOE (%)
V-net^10^	22.99
H-DenseUnet^11^	51.01
PADLLS	12.45

## Discussion

Solid organ segmentation, particularly the liver, has received much attention in recent years and while many reports have now been able to achieve excellent scores for segmentation, there remain multiple challenges for broad adoption of these methods, (1) over/under-segmentation problems still exist, (2) most approaches are still only semi-automated, (3) depending on the underlying architecture of the network, some may perform well at separation of the liver from the heart and diaphragm but poorly with the spleen or bowel, and vice-versa with other networks, (4) most do not make code-bases publicly/freely available, (5) those that are available are often in restricted formats for input/output. We sought to overcome these limitations and provide a deep learning, fully automated liver segmentation pipeline that leverages the strengths of 2D and 3D based learning architectures to result in a model that outperforms the current state of the art models.

DCNN based approaches for liver segmentation have made great advancements in recent years producing multiple strong-performing segmentation models, but each of these models have had different pros and cons to date; no single model has been able to combine the strengths of different network structures (e.g., U-nets versus V-nets). The 2D H-DenseUnet is good at identifying intra-slice features but fails to take into account information along the z-axis, whereas the 3D V-net is good at inter-slice features but has a large computational cost. Furthermore, the majority of the top-performing models require some level of user interface and frequently have different input/output formats, thus limiting the potential for more wide-scale utility by the biomedical imaging community. Here we provide PADLLS as a fully automated pipeline leveraging the benefits of dense V-net and U-nets in addition to heuristic filters to correct oversegmentation problems.

Our pipeline was validated against the 3DIRCADb and SLIVER07 public datasets, which returned average Dice scores of 0.965 and 0.957, respectively; these scores were higher than the H-DenseUnet and V-net. The differences were statistically significant when compared to that of the V-net and H-DenseUnet for both the SLIVER07 and 3DIRCADb challenge. The improvement in performance supports the utility of the pipeline incorporating both network structures. Table 4 compares our pipeline to other segmentation algorithms that participated in the 3DIRCADb challenge in 2017 and some that were published after the challenge. PADLLS outperforms these networks as well. Supplementary Table S3 provides a comparison against additional networks that were only validated against a portion of the 3DIRCADb (including the mU-Net^17^).

**Table 4:** Comparison of other liver segmentation models on 3DIRCADb challenge.

Model	Year	Dataset	VOE (%)	RVD (%)	ASD (mm)	RMSD (mm)	Dice	*P* value
Unet^22^	2017	3DIRCADb	14.21 $\pm$ 5.71	− 0.05 $\pm$ 0.10	4.33 $\pm$ 3.39	8.35 $\pm$ 7.54	0.923 $\pm$ 0.03	0.001*
ResNet^23^	2017	3DIRCADb	11.65 $\pm$ 4.06	− 0.03 $\pm$ 0.06	3.91 $\pm$ 3.95	8.11 $\pm$ 9.68	0.938 $\pm$ 0.02	0.03*
Li et al.^24^	2015	3DIRCADb	9.15 $\pm$ 1.44	− 0.07 $\pm$ 3.64	1.55 $\pm$ 0.39	3.15 $\pm$ 0.98	–	–
Moghbel et al.^25^	2016	3DIRCADb	5.95	7.49	–	–	0.911	–
Lu et al.^26^	2017	3DIRCADb	9.36 $\pm$ 3.34	0.97 $\pm$ 3.26	1.89 $\pm$ 1.08	4.15 $\pm$ 3.1atio6	–	–
U-net + GAN^27^	2018	3DIRCADb	–	–	–	–	0.94	–
Zhang et al.^28^	2020	3DIRCADb	–	–	–	–	0.958	–
DFS U-Net^29^	2021	LiTS	–	–	–	–	0.949 $\pm$ 0.031	–
MSN-Net^30^	2021	LiTS	4.41 $\pm$ 0.06	–	–	–	0.942 $\pm$ 0.01	–
Araújo et al.^31^	2022	LiTS	8.28	− 0.41	–	–	0.9564	–
DALU-Net^32^	2022	Custom	–	–	–	–	0.899 $\pm$ 0.201	–
PADLLS	2022	3DIRCADb	6.66 $\pm$ 2.89	− 0.0421 $\pm$ 0.033	1.31 $\pm$ 0.83	1.94 $\pm$ 1.85	0.965 $\pm$ 0.016	

An asterisk indicates a statistically significant difference in Dice score between our pipeline and another model (*p* < 0.05). A dash indicates inability to assess for statistical significance due to the absence of reported Dice score and standard deviations.

Normal livers have a wide range of morphological configurations (6 classifications according to Netter^18^, with an even larger number based on recent cadaveric studies^19^), thus the potential for oversegmentation by adjacent abdominal organs (particularly the stomach and spleen) is a challenge with current top performing segmentation models. We solved this issue by removing abdominal organs with similar density and close proximity to the liver or touch the liver using the V-net. In this case, we removed the spleen and stomach. These improvements are likely to be even more significant in the analysis of patients with pathological disease or anatomic variations. Abdominal ascites, commonly seen in patients with chronic liver disease resulting in cirrhosis for example, can present a significant challenge for fully automated liver segmentation models. We solved this problem by constructing an initial liver segmentation map, then using an optimized threshold to remove ascites.

Despite the improved accuracy and precision of the pipeline, limitations exist, particularly with respect to scans with significant pathology or post-operative alterations in anatomy. There remain challenges and improvements to be made including addressing pathological aspects of liver disease, such as cirrhotic contours, fatty liver, and mass detection.

One notable limitation of PADLLS is the computational cost incurred due to cascading networks. Once segmentation accuracies exceed 0.9, the incremental computational cost for improvement may be significant. For instance, PADLLS’ modest improvement over Zhang et al’s Dial-3DResUnet in the 3DIRCADb challenge (Table 4), comes with having nearly 80 million learnable parameters relative to approximately 8.4 million in the latter. Most of the computational cost in PADLLS comes from the H-DenseUnet, which has 80 million parameters and takes 64.30 min per case on an 8 GB GPU. In contrast, the V-net only has about 12 million parameters and takes 4.44 s to segment every case. However when we ran PADLLS on the Hoffman2 cluster, segmentation only cost 162 s (32 GB GPU) per case, highlighting the point that over time due to improvements in hardware, the contribution of computational complexity to the overall computational cost decreases, thus the gains for improved accuracy (e.g. better liver segmentation resulting in improved accuracy and diagnostic capabilities for downstream applications) may offset the transiently increased computational costs (that will decrease over time).

In conclusion, we successfully developed a novel liver segmentation pipeline with a plethora of potential applications that are dependent on having a high quality, automated liver segmentation tool. The effectiveness of our pipeline in radiomics and clinical practice ought to be verified in future studies. In the hope that other investigators will benefit from this tool, we have made the source code freely available. We provide a fully automated CT liver segmentation algorithm that combines multiple DCNN architectures and provide it as a freely available tool that we hope will enable further biomedical imaging applications including radiomics^20^ and radiogenomics^21^.

## Supplementary Information

Supplementary Information.

## Abbreviations

ASD: Average symmetric surface distance
BTCV: Beyond the cranial vault
CT: Computed tomography
DCNN: Deep convolutional neural network
HD: Hausdorff distance
kNN: K-nearest neighbor
HF: Hybrid fusion
HU: Hounsfield units
LiTS: Liver tumor segmentation
PADLLS: Pipeline for automated deep learning liver segmentation
RMSD: Root mean square symmetric surface distance
ROC: Receiver operating characteristic
ROI: Region of interest
RVD: Relative volume difference
SLIVER07: Segmentation of the liver competition 2007
VOE: Volumetric overlap error
WL: Window length
WW: Window width
