# Supplementary material for: A pipeline for automated deep learning liver segmentation (PADLLS) from contrast enhanced CT exams
Source: Sci Rep. 2022 Sep 22;12:15794. doi: 10.1038/s41598-022-20108-8 (PMC9500060; doi:10.1038/s41598-022-20108-8)
Supplement: Supplementary file 1 — Supplementary Information. [file 41598_2022_20108_MOESM1_ESM.pdf]

A Pipeline for Automated Deep Learning Liver Segmentation (PADLLS) from Contrast Enhanced CT Exams (Supplementary Materials)

Jayasuriya Senthilvelan and Neema Jamshidi

A. Sub-network architectures

The cascading DCNN model was built upon published, validated networks [9, 11]. We provide a summarized version of these in Supp Tables S1 and S2.

Supplementary Table S1: V-net architecture details*

Layer	Input	Output	Kernel	Stride	Subunits, m x n_f
Feature	144^3 x 1	72^3 x 24	53	2	-
DFS 1	72^3 x 24	72^3 x 20	3^3	1	5 x 4
Skip 1	72^3 x 20	72^3 x 12	3^3	1	-
Down 1-2	72^3 x 20	36^3 x 24	3^3	2	-
DFS 2	36^3 x 24	36^3 x 80	3^3	1	10 x 8
Skip 2	36^3 x 80	36^3 x 24	3^3	1	-
Up 2	36^3 x 24	72^3 x 24	-	-	-
Down 2-3	36^3 x 80	18^3 x 24	3^3	2	-
DFS 3	18^3 x 24	18^3 x 160	3^3	1	10 x 16
Skip 3	18^3 x 160	18^3 x 24	3^3	1	-
Up 3	183 x 24	723 x 24	-	-	-
Up Prior	12^3 x 9	72^3 x 9	-	-	-

*Reproduced from [11], where DFS stands for Dense Feature Stack, m is number of layers, and n_f is number of features in each unit.

Supplementary Table S2: H-DenseUnet architecture details*

	Feature size	2D DenseUNet-167 (k=48)	Feature size	3D DenseUNet-65 (k=32)
input	224 x 224	-	224 x 224 x 12	
convolution 1	112 x 112	7 x 7, 96, stride 2	112 x 112 x 6	7 x 7 x 7, 96, stride 2
pooling	56 x 56	3 x 3 max pool, stride 2	56 x 56 x 3	3 x 3 x 3 max pool, stride 2
dense block 1	56 x 56	[1 x 1, 192 conv; 3 x 48 conv] x 6	56 x 56 x 3	[1 x 1 x 1, 128 conv; 3 x 3 x 3, 32 conv] x 3
transition 1	56 x 56	1 x 1 conv	56 x 56 x 3	1 x 1 x 1 conv
layer	28 x 28	2 x 2 average pool	28 x 28 x 3	2 x 2 x 1 average pool
dense block 2	28 x 28	[1 x 1, 192 conv; 3 x 3, 48 conv] x 12	28 28 x 3	[1 x 1 x 1, 128 conv; 3 x 3 x 3, 32 conv] x 4
transition layer 2	28 x 28	1 x 1 conv	28 x 28 x 3	1 x 1 x 1 conv
	14 x 14	2 x 2 average pool,	14 x 14 x 3	2 x 2 x 1 average pool
dense block 3	14 x 14	[1 x 1, 192 conv; 3 x 3, 48 conv] x 36	14 x 14 x 3	[1 x 1 x 1, 128 conv; 3x3x3, 32 conv] x 12
transition layer 3	14 x 14	1 x 1 conv	14 x 14 x 3	1 x 1 x 1 conv
	7 x 7	2 x 2 average pool	7 x 7 x 3	2 x 2 x 1 average pool
dense block 4	7 x 7	[1 x 1, 192 conv; 3 x 3, 48 conv] x 24	7 x 7 x 3	[1 x 1 x 1, 128 conv; 3 x 3 x 3, 32 conv] x 8
upsampling layer 1	14 x 14	2 x 2 upsampling - [dense block 3], 768, conv	14 x 14 x 3	2 x 2 x 1 upsampling - [dense block 3], 504, conv

upsampling layer 2	28 x 28	2 x 2 upsampling - [dense block 2], 384, conv	28 x 28 x 3	2 x 2 x 1 upsampling - [dense block 2], 224, conv
upsampling layer 3	56 x 56	2 x 2 upsampling - [dense block 1], 96, conv	56 x 56 x 3	2 x 2 x 1 upsampling - [dense block 1], 192, conv
upsampling layer 4	112 x 112	2 x 2 upsampling - [dense block 1], 96, conv	112 x 112 x 6	2 x 2 x 2 upsampling - [convolution 1], 96, conv
upsampling layer 5	224 x 224	2x2 upsampling, 64, conv	224 x 224 x 12	2 x 2 x 2 upsampling, 64, conv
convolution 2	224 x 224	1 x 1, 3	224 x 224 x 12	1 x 1 x 1, 3

*Reproduced from [9]

B. Comparison with state of the art liver segmentation networks

We evaluated the current literature to identify all automated liver segmentation models and tools. Within the main text of the manuscript we provide comparisons to networks that included enough detail for reproduction and direction comparison (i.e. Table 4). There were other networks and published studies that we collected duirng our literature search, but were not about to compare either due to incomplete validation against the entire 3DIRCADb dataset or inaccessibility/availability of the published models. The following models were not discussed in the main text due to incomplete validation of the published studies and/or inability to make a direct comparison in performance by calculation of a quantitative metric.

Supplementary Table S3: Comparison of other liver segmentation models on 3DIRCADb. The 3DIRCADb dataset was composed of 20 CT datasets. In order to provide a fair and complete comparison of calculated DICE results, we believe that the full 20 would need to be used as validation. Only validating against a subset of this (e.g. 3 or 5 CT scans) would likely introduce significant bias (20 CT datasets is already a relatively small sample size).

Model	Year	DICE	n
Deep Belief [1]	2019	0.918 ± 0.137	5
Christ et al. [2]	2017	0.943	3
mu-Net [3]	2020	0.960 ± 0.108	5
Xie et al. [4]	2021	0.961	3
Tran et al. [5]	2021	0.964 ± 0.04	15
DefED-Net [6]	2021	0.966 ± 0.011	5
BATA-Unet [7]	2020	0.967	20
RA-UNet [8]	2020	0.977	20
H-DenseUnet [9]	2018	0.982 ± 0.01	20
Channel U-net [11]	2019	0.984 ± 0.01	20

1. Deep Belief network: Ahmad et al. combined SLIVER and 3DIRCADb datasets. From this list, n = 30 were used for training and n = 10 were used for testing (5 from SLIVER and 5 from 3DIRCADb). Unable to compare to PADLLS because they only tested with 5 of the 20 3DIRCADb CT scans.
2. Christ et al.: Unable to compare to PADLLS because they only tested on 3 of the 20 3DIRCADb CT scans.
3. mu-Net: Unable to compare to PADLLS because they only tested on 5 of the 20 3DIRCADb CT scans.
4. Xie et al.: Unable to compare to PADLLS because Dynamic Adaptive Residual network was only tested on 3 of the 20 3DIRCADb CT scans.
5. Tran et al.: Unable to compare to PADLLS because multiple layer U-net was only tested on 15 of the 3DIRCADb datasets, instead of the entire datasets.
6. DefED-Unet: Unable to compare to PADLLS because this network was only tested on a subset (5 datasets) of the 3DIRCadb datasets, as opposed to a comparison of all of the datasets.
7. BATA-Unet: Unable to compare to PADLLS because Abdalbagi et al. did not make their network available for other researchers.
8. RA-Unet: Unable to compare to PADLLS because Jin et al. did not make their network available for other researchers.
9. H-DenseUnet: Unable to compare to PADLLS because when tested on 3DIRCADb using their code available at https://github.com/xmengli999/H-DenseUNet, we obtained a DICE score of 0.930 ± 0.041, but the score reported in their paper was 0.982 ± 0.01. Also, the reported score includes other datasets in addition to the twenty 3DIRCADb datasets. Additionally, other papers do not agree on what score was achieved by H-DenseUnet. For

instance, Seo et al. in their mu-Net paper claimed 0.951 ± 0.010, Lei et al. in their DefED-Net paper reported 0.957 ± 0.011, but Chen et al. in the original Channel-Unet manuscript there was a reported value of 0.982 ± 0.01.

10. Channel U-net: Unable to compare to PADLLS because Chen et al. did not make their network available for other researchers.

References

1. Gibson E, Giganti F, Hu Y, et al (2018) Automatic Multi-Organ Segmentation on Abdominal CT With Dense V-Networks. IEEE Transactions on Medical Imaging 37:1822–1834. https://doi.org/10.1109/TMI.2018.2806309

2. Li X, Chen H, Qi X, et al (2018) H-DenseUNet: Hybrid Densely Connected UNet for Liver and Tumor Segmentation from CT Volumes. arXiv:170907330 [cs]

3. Ahmad M, Ai D, Xie G, et al (2019) Deep Belief Network Modeling for Automatic Liver Segmentation. IEEE Access 7:20585–20595. https://doi.org/10.1109/ACCESS.2019.2896961

4. Christ PF, Ettlinger F, Grün F, et al (2017) Automatic liver and tumor segmentation of CT and MRI volumes using cascaded fully convolutional neural networks. arXiv preprint arXiv:170205970

5. Seo H, Huang C, Bassenne M, et al (2020) Modified U-Net (mU-Net) with Incorporation of Object-Dependent High Level Features for Improved Liver and Liver-Tumor Segmentation in CT Images. IEEE Trans Med Imaging 39:1316–1325. https://doi.org/10.1109/TMI.2019.2948320

6. Xie X, Zhang W, Wang H, et al (2021) Dynamic adaptive residual network for liver CT image segmentation. Computers & Electrical Engineering 91:. https://doi.org/10.1016/j.compeleceng.2021.107024

7. Tran S-T, Cheng C-H, Liu D-G (2021) A Multiple Layer U-Net, Un-Net, for Liver and Liver Tumor Segmentation in CT. IEEE Access 9:3752–3764. https://doi.org/10.1109/ACCESS.2020.3047861

8. Lei T, Wang R, Zhang Y, et al (2021) DefED-Net: Deformable Encoder-Decoder Network for Liver and Liver Tumor Segmentation. IEEE Transactions on Radiation and Plasma Medical Sciences 1–1. https://doi.org/10.1109/TRPMS.2021.3059780

9. Abdalbagi F, Viriri S, Mohammed MT (2020) Bata-Unet: Deep Learning Model for Liver Segmentation. Signal & Image Processing: An International Journal (SIPIJ) Vol 11:

10. Jin Q, Meng Z, Sun C, et al (2020) RA-UNet: A Hybrid Deep Attention-Aware Network to Extract Liver and Tumor in CT Scans. Front Bioeng Biotechnol 8:. https://doi.org/10.3389/fbioe.2020.605132

11. Chen Y, Wang K, Liao X, et al (2019) Channel-Unet: A Spatial Channel-Wise Convolutional Neural Network for Liver and Tumors Segmentation. Front Genet 10:. https://doi.org/10.3389/fgene.2019.01110
